# Suitability of *Taxodium distichum* for Afforesting the Littoral Zone of the Three Gorges Reservoir

**DOI:** 10.1371/journal.pone.0146664

**Published:** 2016-01-15

**Authors:** Bo Li, Chunlan Du, Xingzhong Yuan, J. H. Martin Willison, Hongyan Xiao

**Affiliations:** 1 Faculty of Architecture and Urban Planning, Chongqing University, Chongqing, China; 2 Postdoctoral Station of Urban-Rural Planning, Chongqing University, Chongqing, China; 3 State Key Laboratory of Coal Mine Disaster Dynamics and Control, Chongqing University, Chongqing, China; 4 School for Resource and Environmental Studies, Dalhousie University, Halifax, Nova Scotia, Canada; 5 School of Chemistry and Chemical Engineering, Yangtze Normal University, Chongqing, China; University of Vigo, SPAIN

## Abstract

The littoral zone ecosystem of the Three Gorges Reservoir (TGR) has become significantly degraded by annual cycles of prolonged winter flooding and summer drought. For purposes of flood control and sediment management, the water level in the reservoir is lowered by 30 m during the summer monsoon season and raised again to 175 m above sea level each year at the end of the monsoon period. To explore an effective way to promote biodiversity and associated ecosystem services, we examined *Taxodium distichum* as a species for afforesting the littoral zone. Sapling growth variations were measured after two rounds of winter flooding. Dominant influence factors were determined by redundancy analysis. Herb community similarities between the experimental afforested areas and nearby control areas were assessed to detect the ecosystem influence of the experimental afforestation. 94.5% of saplings planted at elevations above 168 m survived. All measured growth indices (tree height, diameter at breast height, crown width and foliage density) decreased as the flood depth increased. Completely submerged saplings had a mean dieback height of -0.65 m. Greater initial foliage density led to increased tree height and stem diameter. Shannon-Wiener indices were not significantly different between plots in experimental and control areas, but the low similarity of herb communities between experimental and control areas (0.242 on average) suggested that afforestation would enrich plant community structure and improve littoral zone ecosystem stability. Because littoral zone afforestation provides several ecosystem services (habitat, carbon sink, water purification and landscaping), it is a promising revegetation model for the TGR.

## Introduction

Vegetation is an important element of the functional ecology of reservoir littoral zones and consequent provision of ecosystem services. It improves water quality by filtering agricultural nutrient runoff and trapping sediments [1,2], and reduces flood risks by intercepting runoff and enhancing evaporation via transpiration [3,4]. Littoral zone plants can reduce soil erosion and reinforce bank stability [5]. Vegetation communities in the littoral zone provide habitat for fish [6], birds [7] and biodiversity in general. In light of these benefits, restoring vegetation to damaged reservoir ecosystems is widely regarded as important [8,9].

The Three Gorges Dam (TGD) constructed on the Changjiang (Yangtze River) at Yichang City in China created the Three Gorges Reservoir (TGR) which runs more than 600km upstream to Jiangjin County of Chongqing [10]. The TGR management strategy is popularly expressed as “storing clear water and releasing muddy”: the water level is drawn up to 175 m above sea level in winter and is drawn down to 145 m above sea level in summer. On the one hand, this strategy has provided the benefits of flood control, hydropower generation, improved navigation and sediment release; on the other hand, the 30 m water level fluctuation has produced a huge littoral zone which is largely bare of vegetation. Many plant species have disappeared from the littoral zone due to lack of adaptation to prolonged winter flooding followed by extended summer drought, and ecosystem functions such as maintenance of plant cover and resistance to erosion have been significantly degraded as a result [11–13]. Some ruderal plant species have propagated rapidly in the altered littoral zone of the TGR (LZTGR), such as outbreaks of *Xanthium sibiricum* in 2008 and 2009 [14]. For these reasons, it is important to find appropriate methods for improving ecological functions and services in the bays and minor tributaries of the reservoir.

Ecosystem restoration by rehabilitating vegetation in the LZTGR has been extensively studied in recent years. One group of perennial non-woody plants (*Acorus calamus*, *Alternanthera philoxeroides*, *Arundinella anomala*, *Hemarthria compressa*, *Triarrhena sacchariflora*, *Vetiveria zizanioides*, *Cynodon dactylon* and *Cyperus rotundus*) has been identified as suitable for combating soil erosion [15,16], and a group of agricultural hydrophytes (*Nelumbo nucifera*, *Sagittaria trifolia* var. *edulis*, *Eleocharis dulcis*, *Zizania latifolia*, *Trapa bispinosa* and *Oryza sativa*) has been reported as promising species for alleviating the conflict between ecosystem conservation and agricultural production [17]. In addition, some woody species such as *Salix variegata* [18], *Salix rosthornii* [19], *Distylium chinense*[20], *Populus x canadensis* [21], *Pterocarya stenoptera* [22], *Morus alba* [23], *Nyssa aquatica* [24], *Taxodium ascendens* and *Taxodium distichum* [25] have been examined as candidate species for revegetation of the LZTGR. Of these, *Nyssa* and *Taxodium* are native to the United States, *Populus* species are widespread in China (*Populus x canadensis* is a horticultural hybrid), and the other species are native to China.

*T*. *distichum* was first introduced into regions of China having suitable humid monsoon climatic conditions at least 100 years ago and is now used widely in wetland landscaping. The species has excellent flood tolerance in the littoral zones of reservoirs in the United States [26,27], and although it has been proposed as a promising candidate for revegetation projects in the LZTGR [25], studies reported to date have been conducted in greenhouses [28,29] or in regions of the TGR mostly subjected to summer flooding [30]. Experiments are needed on the acclimation, performance, and habitat effects of *T*. *distichum* seedlings and saplings in typical water-fluctuation conditions of the LZTGR itself.

In this paper we report experimental afforestation with *T*. *distichum* conducted in the context of an ecological engineering model for improving ecosystem functions in the LZTGR. The model is derived from field experiments and named 'littoral woods engineering' (LWE) [12]. We have addressed the following questions. 1) To which water-fluctuation elevation ranges are saplings of *T*. *distichum* adapted in the TGR, and how well do they perform at these elevations? 2) How does the status of *T*. *distichum* saplings prior to submersion affect their subsequent performance in the LZTGR? 3) What are the effects of this afforestation on the understory herb communities and on LZTGR ecosystem stability?

## Materials and Methods

### Site description

**Fig 1:**
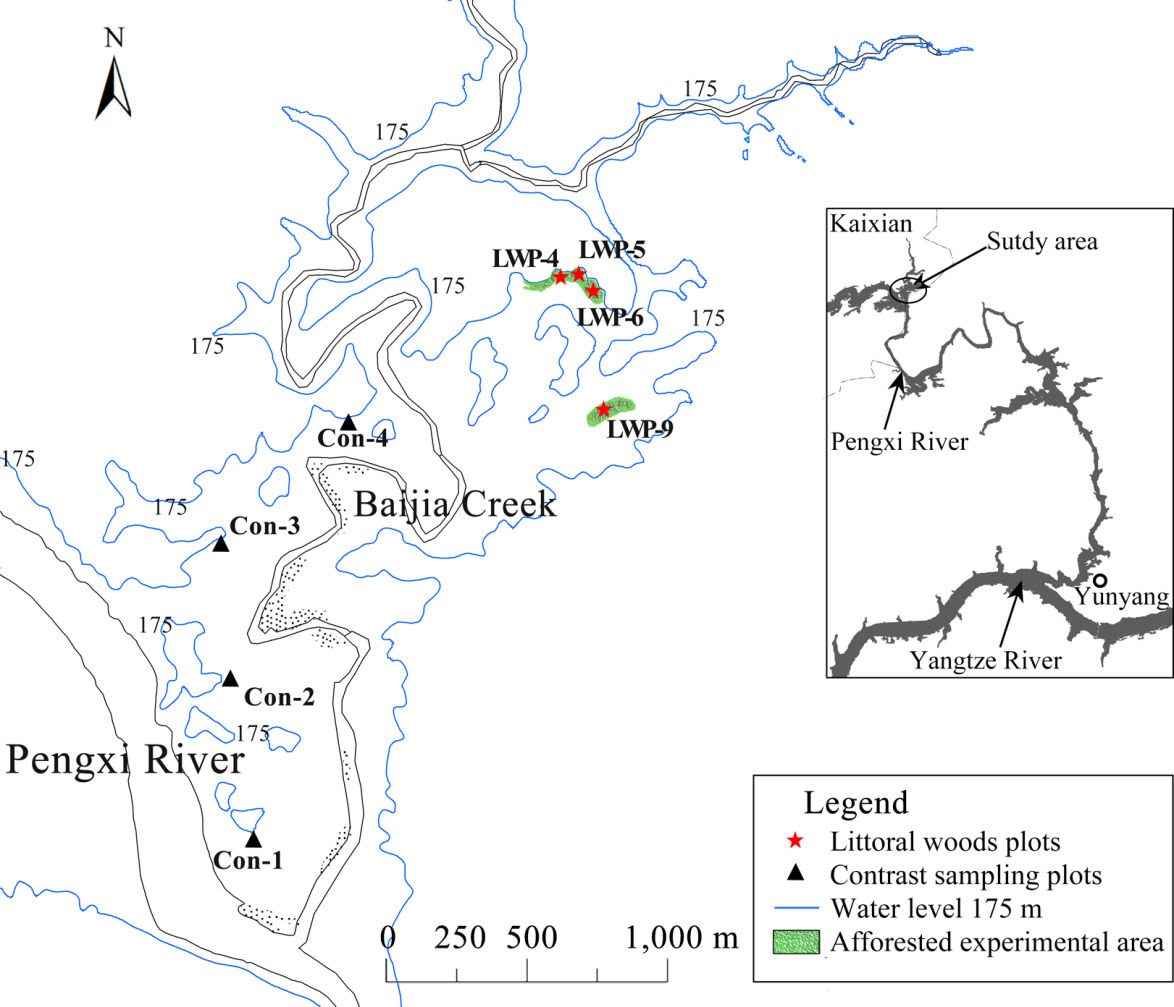
Locations of the afforested experimental plots and control plots. The study site is adjacent to the Pengxi River, a tributary that lies north of the middle reaches of the Changjiang. The afforested experimental plots were distributed from 168 m to 173 m above sea level.

The study site lies in the experimental zone of Pengxi River Wetland Natural Reserve, on the banks of the Baijia Stream associated with the Pengxi River, a tributary of the middle reaches of the Changjiang, lying to its north. The experimental zone was expressly established to permit and encourage experiments of the sort described in this paper. The planting site is about 291 km upstream from the TGD, close to a small research station which was set up to facilitate research on the reservoir drawdown zone (latitude 31°09′ N, longitude 108°34′ E, Fig 1). The China Three Gorges Corporation implemented land clearing in the LZTGR when the TGD was under construction and almost all the trees and shrubs were removed from the LZTGR [31], including this site. Because the study site is directly affected by seasonal water-level fluctuation of the TGR, it is characterized by prolonged winter flood and extended summer drought. As a result, many pre-existing local plants have not adapted to the alternating flood-drought environment and have disappeared [12]. Like other regions in the LZTGR, the ecosystem of the study area has been characteristically unstable since 2008 when the water level was first raised to more than 173 m above sea level [11].

### Plant material

On March 28th, 2009, 168 four-year-old *T*. *distichum* trees, grown from seed, were obtained from Lushan Seedling Company in Jiujiang City, Jiangxi Province, China. The average tree height (TH) was about 2.5 m and average diameter at breast height (DBH) was about 2.0 cm. With the permission of Pengxi River Wetland Natural Reserve Management Bureau, all the trees were planted in the study area 4 days after being dug from the donor field. Each was transplanted with an attached earth ball, about 30 cm in diameter.

### Experimental design

Nine fallow fields lying at elevations between 168 m and 173 m above sea level were established as experimental plots for planting the *T*. *distichum* trees (Fig 1). The 168 trees were planted with 4-m spacing between individuals and numbered sequentially. The plots were in two clusters and were selected to be as similar as possible to each other with respect to physiographic conditions and soil conditions, with the intent that two sets of variables could be monitored: hydrological variables due to elevation among the plots, and the variable initial biological conditions of the trees. Statistical and ordination analyses were conducted to determine the dominant factors that influenced tree growth using an experimental approach similar to that of Jimenez et al. [32] and Li et al. [33]. To detect possible effects of *T*. *distichum* afforestation on understory vegetation, we surveyed the herb communities in 4 plots within the afforested experimental areas, and a set of 4 control areas lying at similar elevations within the Baijia Stream drawdown zone nearby (Fig 1). The control areas had not been influenced by the afforestation work. The understory investigation was conducted about 30 months after the plantations had been established.

### Sampling and data analysis

**Fig 2:**
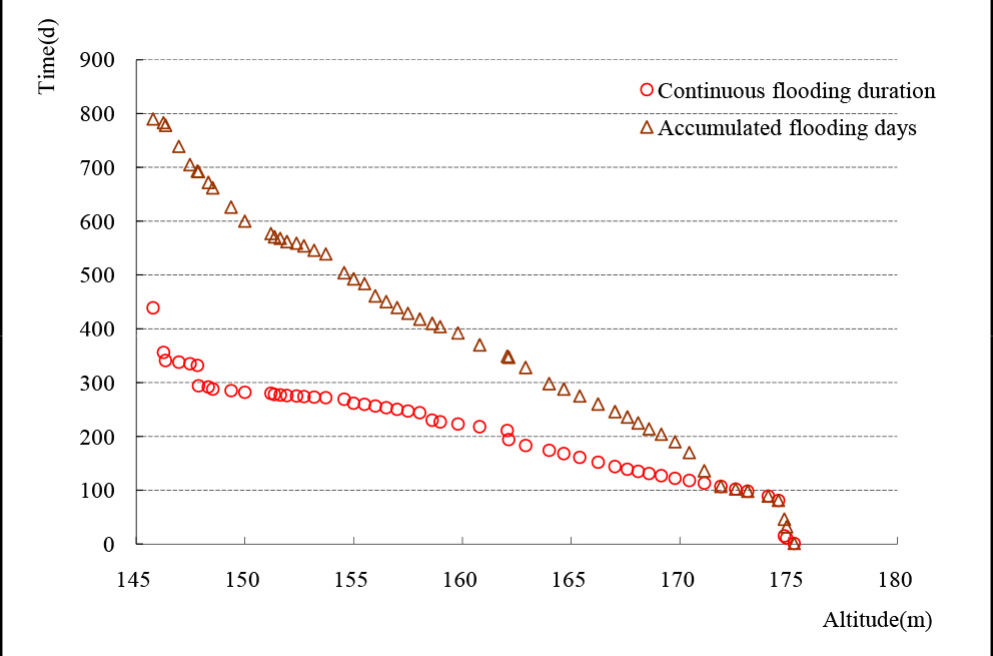
Flooding conditions at different altitudes during July 1, 2009 to August 31, 2011. The duration of continuous flooding was more than four months at 168 m above sea level, and there were about 225 accumulated days of flooding at that elevation.

The following growth indices were measured on each tree in August 2009 and August 2011: TH, DBH, tree top elevation (TTE, the sum of plot altitude and TH), living crown width (CW, mean value measured in two opposite directions), and foliage cover (FC). For FC we measured the proportion of sky occluded by foliage when looking up at the canopy from beneath the tree [34]. This was done on a per tree basis by the same observer. Hydrologic data from the Wanzhou station (about 289 km upstream from the TGD) were recorded continuously and used to estimate the water level variation of the study site (Fig 2).

Surveys of understory vegetation were conducted in late August and early Sept 2011. In the afforested experimental area, 3 square sample plots of 1 m^2^ were set randomly in each of the 4 largest experimental plots (Table 1, LWP-4, LWP-5, LWP-6, LWP-9, containing 116 trees in total), and for comparison 4 sampling sites containing the characteristic dominant plant communities of the contrast region were selected and 3 square sample plots of 1 m^2^ were examined within each of the 4 sampling sites. Number of individuals, height, and coverage of each plant species in each sample plot were recorded separately.

**Table 1:** Basic information about littoral woods plots.

Plot	Average altitude (m)	Seedling number	Flooding duration(d)		Flood depth(m)	
			2009–2010	2010–2011	2009–2010	2010–2011
LWP-1	170.76	10	45	115	0.79	4.49
LWP-2	171.48	11	5	109	0.07	3.77
LWP-3	173.24	9	0	96	0.00	2.01
LWP-4	172.80	26	0	99	0.00	2.45
LWP-5	171.53	29	2	109	0.02	3.72
LWP-6	172.40	25	0	103	0.00	2.85
LWP-7	169.14	10	78	127	2.42	6.12
LWP-8	170.35	12	54	118	1.20	4.90
LWP-9	170.13	36	60	119	1.42	5.12

The redundancy analysis (RDA) method [35] was used to evaluate the growth responses of the trees to the following influence factors: TH, TTE, DBH, CW, FC, altitude (Alt), continuous flooding duration (FLD) and accumulated flooding duration (Acc-FLD).

Species diversities of herb communities were characterized by Richness index *S*: *S = s* (total number of species), and Shannon-Wiener diversity index *H’* [36]: $H'=\sum_{i=1}^{s}\left(p_{i}\right)\left({\text{log}}_{2}\;p_{i}\right)$, where *p*_*i*_ is the proportion of the *i*th species. Plant communities were distinguished according to importance values (*IV*) [37,38]: *IV* = (*Dr* + *Cr* + *Hr*)/3, where *Dr*, *Cr* and *Hr* are respectively the relative density, coverage and height of the *i*th species in sampling plots. A symmetric square matrix of similarities among sampling plots was constructed using the abundance-based Bray-Curtis coefficient: $S_{jk}=1-\sum_{i=1}^{n}\left|y_{ij}-y_{ik}\right|/\sum_{i=1}^{n}\left(y_{ij}+y_{ik}\right)$, where *y*_*ij*_ (or *y*_*ik*_) is the abundance of the *i*th species in the *j*th (or *k*th) sample [39]. The RDA ordination was conducted using the CANOCO software package (version 4.5) [40], while other statistical analyses were performed with SPSS 17.0 software.

## Results

### Growth status

**Fig 3:**
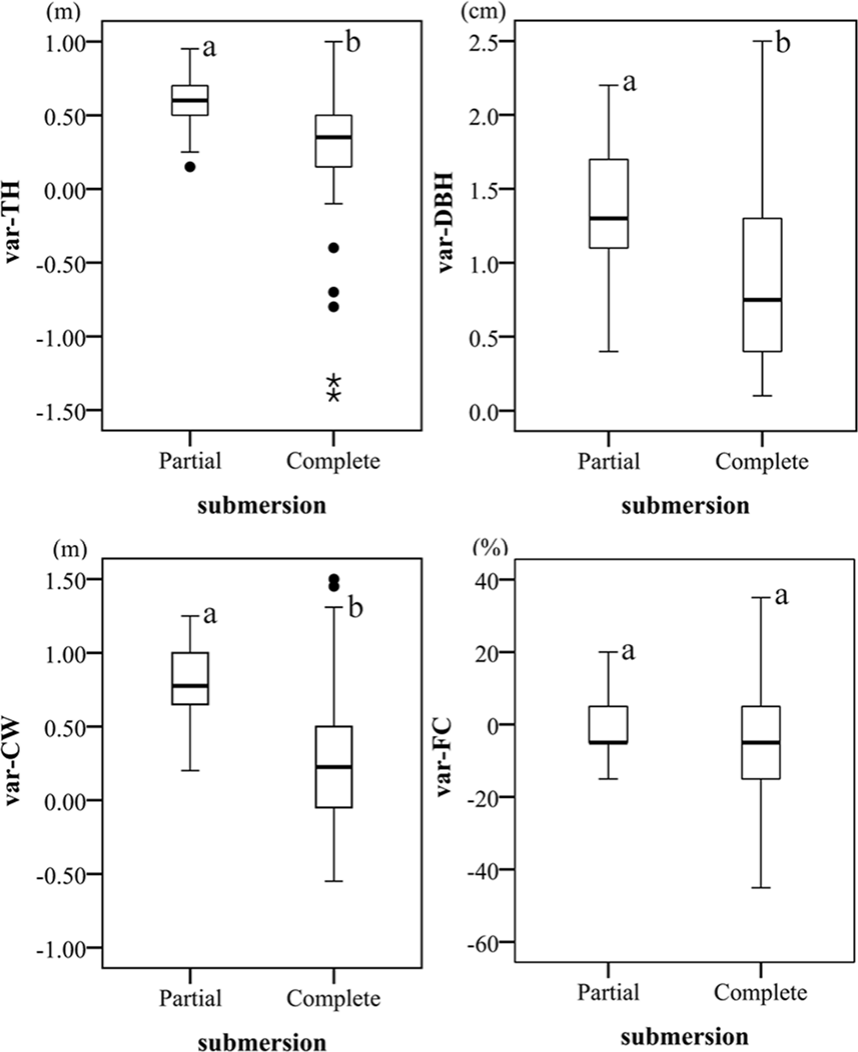
Variations of TH, DBH, CW and FC under partial and complete submersion conditions. Significant differences between the two conditions were analyzed using the Mann-Whitney U test. Box plots with different letters (a, b) were significantly different at the 0.05 level. "·" indicates data considered mild outliers, and "*" indicates data considered extreme outliers.

In August 2009 (about 5 months after transplantion), 163 trees had survived (survival rate 97.02%), with average parameters: TH 2.68 m, DBH 2.11 cm, CW 0.76 m, and FC 78.16%. Two years later, in August 2011, 154 trees had survived after two rounds of submersion (survival rate of 94.48%), with the following changes in parameters: TH 0.37±0.03 m, DBH 0.96±0.05 cm, CW 0.37±0.04 m and FC 4.28±1.26%. There were significant differences (Mann-Whitney U Test, p<0.05) between trees in partly and completely submerged situations with respect to variation of TH (var-TH), DBH (var-DBH) and CW (var-CW), but not of FC (var-FC) (Fig 3). In 2011, surviving trees that had been completely submerged had dieback rate of 6.35%, and mean dieback height (measured from top of dead stem to top of living tissue) of -0.65 m.

### RDA ordination (growth response)

Results of Monte Carlo permutation tests showed highly significant effects (*p* = 0.002 with 499 permutations) of the influence factors on the growth responses of the *T*. *distichum* trees. As shown in the RDA summary (Table 2), the influence factors explain about 26.4% of the total variation of plant growth, and the first two axes taken together displayed most (91.4%) of the variation.

**Table 2:** Summary of the RDA analysis of *T*. *distichum* sapling growth responses to influence factors.

Axes	1	2	3	4	Total variance	P-value
Eigenvalues	0.139	0.102	0.020	0.003	1	
Species-environment correlations	0.521	0.679	0.366	0.147		
Cumulative percentage variance						
of species data	13.9	24.1	26.1	26.4		
of species-environment relationships	52.8	91.4	99.9	100		
Sum of all eigenvalues					1	
Sum of all canonical eigenvalues					0.264	
Test of significance of all canonical axes*						0.002

*Monte Carlo permutation test with 499 permutations was selected for the significance test of all canonical axes.

**Fig 4:**
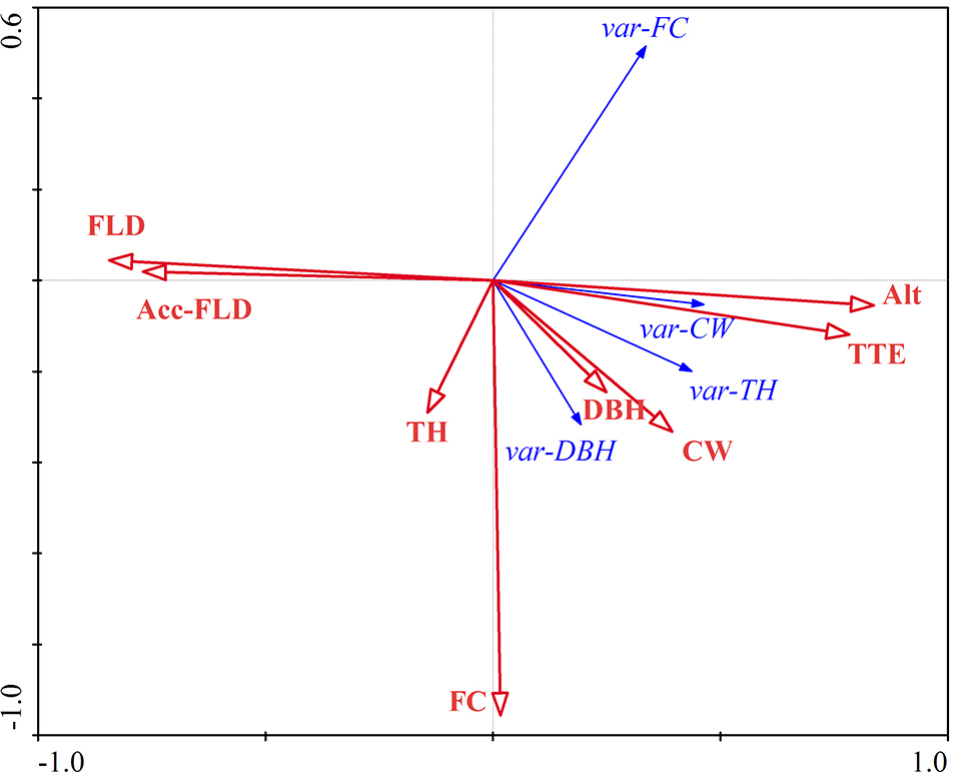
Growth variation-influence factor biplot diagram from the RDA. Growth variations are shown as arrows with solid arrowheads, and influence factors are shown as arrows with empty arrowheads. The approximate correlation between growth variation and influence factor is equal to the cosine of the angle between the corresponding arrows.

The growth variation influence-factor biplot diagram derived from the RDA ordination (Fig 4) showed that the dominant influence factors on tree growth were FC, FLD, Acc-FLD, Alt and TTE (positively correlated with Alt, r = 0.9743). Foliage cover (FC) was positively correlated with variations in var-TH and var-DBH. The Alt variable was positively correlated with all the growth parameters, especially var-CW. By contrast, the flood variables (FLD and Acc-FLD) were negatively correlated with all growth parameters. The morphological variable, FC, was positively correlated with var-DBH and var-TH, negatively correlated with var-FC, and almost uncorrelated with var-CW.

### Herb communities

Shannon-Wiener (*H’*) and species richness (*S*) indices of biodiversity are summarized in Table 3. *H’* indices of understory herb communities in the afforested experimental area were distributed within the range 1.474±0.172–2.652±0.201, in contrast with the range 1.814±0.476–2.526±0.264 in control plots. The *S* indices in the afforested experimental areas were 4.000±0.577–8.667±0.882, in contrast with 5.000±1.155–9.667±0.667 in control plots. Results of One-Way ANOVA analysis indicated that there were no significant differences in *H’* between most plots, with the exception of plot LWP-9 compared with plots LWP-4 and Con-3, but there were more plots which had significant differences in species richness (S), such as plot Con-1 and Con-3, Con-1 and LWP-4, Con-4 and LWP-9.

**Table 3:** Herb community diversity among plots

Plot	Shannon-Wiener (*H’*)	Richness (*S*)
	Mean±S.E.	Mean±S.E.
Con-3	2.526±0.264^a^	8.667±0.882^ab^
Con-1	1.814±0.476^ab^	5.000±1.155^cd^
Con-4	2.268±0.092^ab^	9.667±0.667^a^
Con-2	1.824±0.326^ab^	8.333±0.882^ab^
LWP-4	2.625±0.201^a^	8.667±0.882^ab^
LWP-5	1.763±0.125^ab^	6.667±0.667^bcd^
LWP-6	1.880±0.39^ab^	7.000±1.155^abc^
LWP-9	1.474±0.172^b^	4.000±0.577^d^

^(a,b)^ Means followed by a different superscript (a, b, c, d) are significantly different at the 0.05 level.

Eight herb species were found only in the afforested experimetal area, while 12 species were found only in the control plots. The *IV* indices of species in each plot were calculated and a similarity matrix of *S*_*jk*_ among sampling plots was constructed for further analysis of community structures. According to the *IV* numbers in Table 4, the principal plant communities in the afforested experimental area were the *Cynodon dactylon*+*Bidens pilosa*, *Oplismenus compositus*, *Setaria viridis* and *Bidens pilosa* associations. By contrast, the most widespread plant communities in control plots were the *Cynodon dactylon*, *Xanthium sibiricum*+*Cynodon dactylon*, *Conyza japonica*+*Bidens pilosa* associations. The similarity matrix (Table 5) indicated that communities in the control plots were very dissimilar to most of those in the plots of the afforested experimental area (with the exception of plot LWP-5). Similarities between herb communities in the control plots were generally higher than those of experimental plots.

**Table 4:** Importance values of herbaceous plant species in the plots

plant species	Con-1	Con-2	Con-3	Con-4	LWP-4	LWP-5	LWP-6	LWP-9
*Aeschynomene indica*	4.906	4.789	12.978	1.334	8.043		5.314	9.573
*Alternanthera philoxeroides*			1.159		6.450			
*Alternanthera sessilis*			7.129					
*Amaranthus tricolor*	2.368			2.089				
*Artemisia carvifolia*		1.867		3.428				
*Arthraxon hispidus*						7.024	9.327	
*Avena fatua*				3.239				
*Bidens pilosa*		21.712	4.372	11.125	25.156	20.125	16.018	
*Centella asiatica*		1.037						
*Coix lacryma-jobi*							3.896	
*Conyza japonica*		23.931	9.278	8.596		4.923		16.328
*Cynodon dactylon*	24.973	18.425	24.056	19.817		24.298		
*Cyperus iria*				1.044				
*Cyperus nipponicus*							11.639	
*Cyperus rotundus*		1.677		3.822	6.096		5.438	
*Digitaria sanguinalis*	8.170		5.703	13.778	4.792	14.784	3.310	23.062
*Echinochloa crusgalli var*. *zelayensis*	17.737	2.196	9.889		6.683	5.553	4.440	
*Eclipta prostrata*	4.030		6.972	1.220				
*Imperata cylindrica*		2.473				2.867		
*Ixeris polycephala*								6.863
*Leonurus artemisia*		3.493						
*Oplismenus compositus*					3.087	6.189	25.260	
*Oxalis corniculata*		1.680						
*Patrinia villosa*		4.398						
*Phyllanthus urinaria*					7.592	1.324	1.256	
*Plantago asiatica*			0.888					
*Polygonum hydropiper*	8.740		7.259	4.165	5.093		5.785	
*Polygonum perfoliatum*		4.299		4.985			4.000	
*Rabdosia amethystoides*					4.602			
*Ranunculus sieboldii*			1.391		7.426			
*Salvia plebeia*					2.101			
*Setaria viridis*	3.527	3.003	6.462	14.854	5.626	12.915	4.316	37.534
*Solanum nigrum*				3.785				
*Torilis scabra*		5.019		2.720				6.639
*Xanthium sibiricum*	25.548		2.464		7.253			
**Total**	**100**	**100**	**100**	**100**	**100**	**100**	**100**	**100**

**Table 5:** Similarity index matrix of herb communities in the plots

	Con-1	Con-2	Con-3	Con-4	LWP-4	LWP-5	LWP-6	LWP-9
**Con-1**	1.000	0.400	0.525	0.432	0.176	0.366	0.075	0.044
**Con-2**	0.400	1.000	0.383	0.407	0.193	0.398	0.106	0.202
**Con-3**	0.525	0.383	1.000	0.533	0.210	0.493	0.063	0.154
**Con-4**	0.432	0.407	0.533	1.000	0.168	0.639	0.141	0.444
**LWP-4**	0.176	0.193	0.210	0.168	1.000	0.240	0.262	0.037
**LWP-5**	0.366	0.398	0.493	0.639	0.240	1.000	0.211	0.277
**LWP-6**	0.075	0.106	0.063	0.141	0.262	0.211	1.000	0.011
**LWP-9**	0.044	0.202	0.154	0.444	0.037	0.277	0.011	1.000

## Discussion

### Growth variation of *Taxodium distichum*

*T*. *distichum* is native to the southern region of the United States. Within its natural range, it has long been a component of the mix of arboreal species selected for projects designed to restore ecological functions to forest-depleted bottomlands [41] and damaged ecosystems such as mine sites [42]. It has also been planted in littoral zones of artificial reservoirs, both within its native range [43] and beyond its native range in the western region of the United States [44]. *T*. *distichum* is adapted to specific natural flood pulse conditions which limit its capacity to establish self-sustaining populations [45]. Natural recruitment has been reported to occur following prolonged reservoir drawdown [26], but not in the absence of drawdown [27]. As a result of limited recruitment capacity, the species has not invaded beyond its natural range and even within its native habitat can be difficult to establish as naturally regenerating populations because the optimal conditions for natural spread may not occur annually [46]. Although it has been grown for many years in China, it has not become invasive and is not listed as an invasive species of concern [47].

While *Taxodium distichum* is well recognized as a flood-tolerant species in its native habitat, the environmental conditions in the southeastern United States are not similar to those in the LZTGR, which is characterized by unusually prolonged winter flooding, often followed by extended summer drought. Despite the difference in environment, *T*. *distichum* performed well in the experiment reported here. According to previous reports, *T*. *distichum* seedlings tolerate complete submergence for no more than a month during the growing season [48,49], but in our study transplanted saplings survived at least 3 months of continuous flooding, some of which were completely submerged.

We found that *T*. *distichum* survived in the LZTGR at elevations above 168 m. As shown in Fig 3, the growth parameters (TH, CW and DBH) of all partly-submerged saplings (i.e. having tops above the water surface throughout the winter flood period) increased, despite a tendency for foliage cover (FC) to decline. By contrast, completely submerged saplings grew more slowly on average (Fig 3). The TH and CW of some individuals declined due to die-back from the tip (Fig 3). Greater depth and period of flooding led to slower sapling growth rates in the LZTGR (Fig 4), which corresponds with findings for growth of *T*. *distichum* at the margins of the Mississippi River [50]. Although the average growth rates of completely submerged saplings were lower than partly submerged saplings, a few exceptional individuals were apparently stimulated by submergence (Fig 3).

Analysis revealed that the status of the *T*. *distichum* saplings before they were flooded significantly affected subsequent variation in their growth. Although var-FC was negatively correlated with FC, it appears that greater FC led to better sprouting and enhanced photosynthesis, which in turn led to better assimilation of materials and energy, greater TH and DBH, and therefore greater ability to overcome flooding in the long term by means of the tree top rising above flood water.

Flood tolerance by *T*. *distichum* has been attributed to growth in diameter and the formation of aerial roots and knee roots [51,52]. In our experiment, few aerial roots and knee roots were observed. The reason for this is not clear, but Yamamoto [51] found that knee roots form in an alternating (rather than an extended) state of oxidation and reduction. Ethylene that is produced and accumulates in the plant under anoxic conditions promotes increase of stem diameter and the development of aerenchyma [53], which permits transportation of oxygen from the atmosphere to the roots [54], thus helping flood-tolerant plants to cope with anaerobiosis.

### Influence of afforestation on the understory herb communities

Previous investigation of the vegetation in the LZTGR suggested that plant communities at the same altitude range in any local area are similar [55]. For this reason, we compared the understory herb communities of the afforested experimental plots with herb communities at the same elevation in nearby unmanaged areas.

Average plant species richness per unit area (1 m^2^) was less than 10 in both the afforested experimental area and the control area. The perennial grass *Cynodon dactylon* was a dominant species in all of the control plots, but annual plants (*O*. *compositus*, *S*. *viridis*, *B*. *pilosa*, *D*. *sanguinalis*) were dominant in most of the experimental plots, with the exception of LWP-5. In previous studies, *C*. *dactylon* has been recommended as a flood-tolerant species that is useful for alleviating soil erosion in the LZTGR and improving its aesthetic appearance [56]. *C*. *dactylon* is clearly valuable for ecological restoration of the LZTGR where trees are not suitable, notably in the main transportation corridor of the reservoir, but a diversity of revegetation approaches is more appropriate in the many bays and minor tributaries of the reservoir. In these bays and minor tributaries, soil erosion is of less concern and sediment often accumulates during the winter flood periods, leading to colonization of the LZTGR by ruderal weeds when the water level falls [14].

Prolonged flooding not only affected the vitality and growth rate of the saplings in the experiment, but also brought about changes in the understory community. This finding is consistent with reports on stand composition and structure in typical forested wetlands [57], but drought tolerance may be more significant than flood tolerance in determining which annual plants dominate, and reproduce within, the understory communities. Successful colonists may have seeds that can be dispersed via water [58], lie dormant through the winter flood period, and germinate quickly once flood water recedes. The dispersal characteristics of species are important considerations for effective plant establishment [59] and should be taken into account when selecting candidate species for revegetation of the LZTGR.

### Potential functions of afforestation

**Fig 5:**
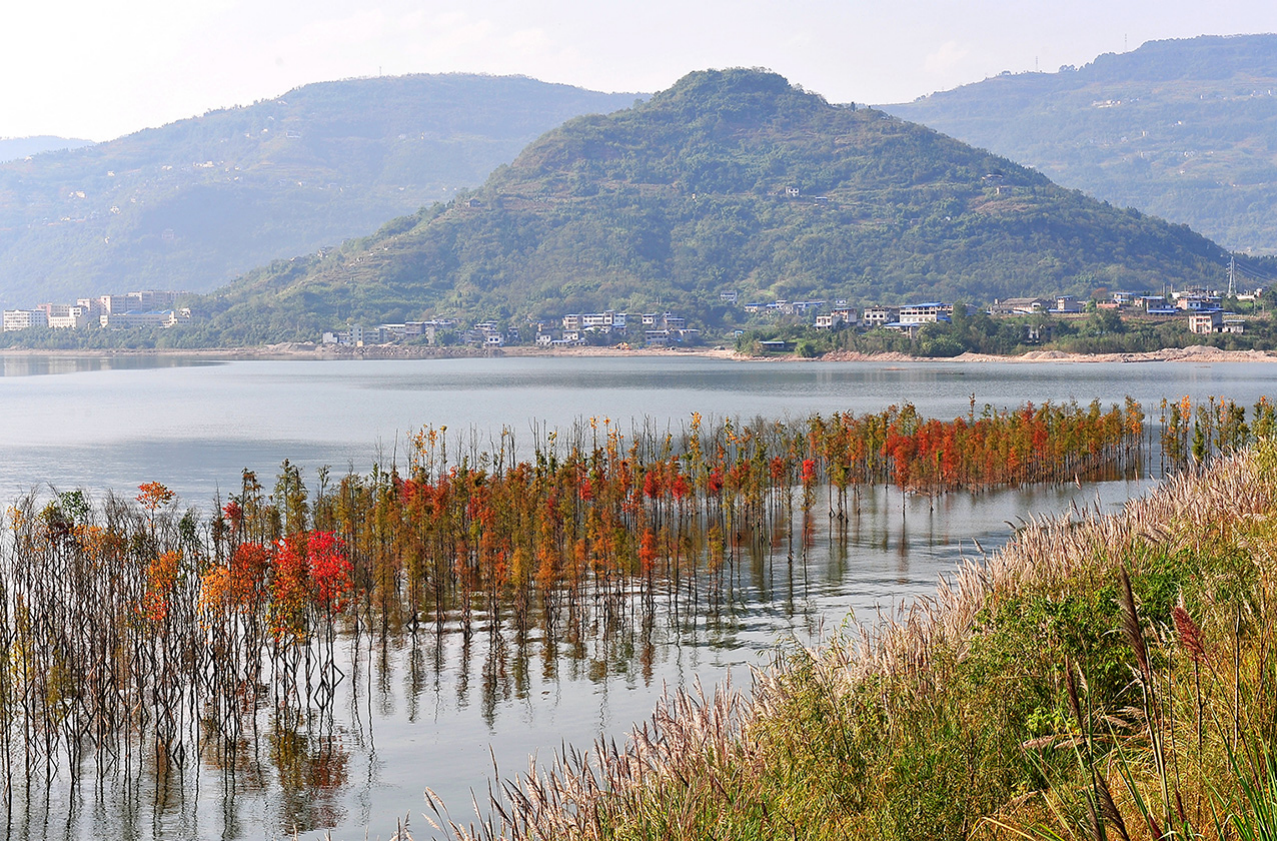
LWE of the littoral zone of Hanfeng Lake in Kaixian town, Chongqing Municipality (photographed by Kang Liu on Nov. 14, 2014). Afforestation has enriched the waterfront landscape in winter.

Afforestation of the LZTGR has many potential functions in improving the ecosystem quality. It could provide habitat for various animals living in and around the littoral zone [60]. Firstly, almost all the shrubs and trees in the LZTGR were removed when the TGD was built, but through afforestation we can try to reintroduce well-adapted trees and shrubs and in that way rebuild plant community structures and improve habitat quality [61]. Littoral zone afforestation may provide habitat for resident birds [62] and create foraging and sheltering places for migratory waterfowl in winter. Secondly, littoral woods can act as carbon sinks when carbon is fixed as organic matter in the form of root, bole and branch through photosynthesis [63–65]. Thirdly, littoral woods with understory plant communities create a barrier which can intercept non-point source pollutants carried by upland surface runoff. Lastly, afforestation can improve the aesthetic appearance of the LZTGR, which would be popular in the many towns situated along the banks of the reservoir (Fig 5).

### Proposals and recommendations

The study reported here was an initial attempt to carry out LWE in the LZTGR. In order to properly understand the potential role of afforestation in restoring LZTGR ecosystems, further research is required to optimize afforestation, as follows.

1. Afforestation influences the understory vegetation [66], and it is reasonable to expect an effect in the case of littoral zone, but by what mechanisms does afforestation affect plant community structure in the understory?
2. Sustained attention and long term monitoring are needed to discover whether saplings that died back (in the experiment described here) will survive in the long term. When selecting and testing the application of other flood-tolerant candidate species, testing for tolerable flood-depth ranges should be conducted in the field using well-designed experimental protocols [57].
3. Several herb, shrub and tree species have been shown to tolerate flooding in the LZTGR, such as *Cynodon dactylon* [67], *Vetiveria zizanioides* [15], *Calamagrostis arundinacea*[68], *Salix variegata* [18], *Salix rosthornii* [19], and “Zhongshanshan 118” (a *Taxodium* hybrid used in silviculture) [69]. It will be useful to construct experimental complex littoral-woods ecosystem models composed of trees, shrubs and grasses, and test these under field conditions.
4. It may be argued that the leaf litter from littoral woods would decompose under water and lead to net release of greenhouse gases. Would this be the case? Mitsch et. al. [70] suggested that most wetlands are net carbon sinks, even when greenhouse gas emissions were considered. In the LZTGR, leaf litter is generated mainly in winter and the relatively low water temperature will decrease methanogen activity. For this reason, the amount of greenhouse gases emissions resulting from littoral zone afforestation is uncertain. Littoral woods are complex systems: on one hand, some greenhouse gases will be emitted, but on the other hand woody plant growth will lead to storage of atmospheric carbon in the form of biomass [71,72]. Furthermore, litter fall into the TGR ecosystem will contribute dissolved organic carbon and nutrients to the TGR ecosystem [73,74], and these play important roles in biogeochemical reactions while also supplying nutrients and energy to microbial food webs [75,76]. Therefore, the advantages and disadvantages of littoral zone afforestation in general need further experimentation and modelling.
5. Since water-level variation is the dominant factor affecting plant community structure in the LZTGR, is it possible to create a more robust littoral-zone ecosystem by adjusting the water-level regulatory regime of the Three Gorges Reservoir? The Glen Canyon Dam Adaptive Management Program and the Missouri River Recovery Program [77,78] provide examples of achieving a balance between environmental protection and water resource development by means of adaptive management and stakeholder engagement.

## Conclusions

The results of this research indicate that the *T*. *distichum* saplings had excellent tolerance of winter flooding, with survival rate of more than 94% after two rounds of annual winter flooding when planted at elevations of 168m and higher above sea level. All the growth indices measured (CW, TH, DBH and FC) decreased as the flood depth increased. The growth of the *T*. *distichum* saplings in the LZTGR was also significantly influenced by their status prior to submersion, particularly FC.

The low levels of plant biodiversity in the control plots suggests that ecosystem restoration via natural recolonization is insufficient for rapidly restoring ecosystem health to the LZTGR and that interventions, such as afforestation, are indicated. Although there were no significant differences between most *H’* indices of the herb-community plots in the *T*. *distichum* afforested experimental area and control area, similarity analysis indicated that herb community complexity was increased by the afforestation, and could therefore be helpful for enhancing the stability of the LZTGR ecosystem. We conclude that the afforestation method described here has potential for improving ecosystem quality, but that further experimental research is required before afforestation with *T*. *distichum* can be proposed for wide application in the LZTGR. This experimentation might include assessment of the potential for *T*. *distichum* to establish self-sustaining populations (which could be beneficial), or to become invasive beyond the sites for which it has been selected (which could be deleterious). The economic acceptability of *T*. *distichum* for local people, notably farmers, should also be examined. Continued monitoring of the biological communities that establish in association with populations of *T*. *distichum*, both at this site and others in the LZTGR, should continue so that its ecological functions can be properly understood. Littoral zone afforestation experiments involving the creation of multi-aged multi-species woods that more closely mimic natural populations are clearly indicated, as are experimental afforestations of diverse sites which vary with respect to environmental conditions such as soil, slope, aspect, and hydrological variables.

## Supporting Information

S1 Table Water level records of Wanzhou Sation during July 1, 2009 to August 31, 2011. (XLS)

S2 Table Species abundances in the sampling plots. (XLS)
